# Traumatic spondyloptosis of the lumbar spine: a case report

**DOI:** 10.1186/1752-1947-8-453

**Published:** 2014-12-21

**Authors:** Robert Amesiya, Norbert Orwotho, Mallon Nyati, Rodney Mugarura, Erisa Sabakaki Mwaka

**Affiliations:** 5 Orthopaedics department, School of Medicine, Makerere University, P.O. Box 7072, Kampala, Uganda; 6 Orthopaedics department, Mulago Hospital, P.O. Box 7051, Kampala, Uganda; 7 Anatomy department, School of Biomedical Sciences, Makerere University, P.O. Box 7072, Kampala, Uganda

**Keywords:** Lumbar, Neurological improvement, Posterior instrumentation, Spinal cord injury, Spondyloptosis

## Abstract

**Introduction:**

Spondyloptosis is the most severe of translation spine injuries. It results in complete disruption of the structural elements of the vertebral column and the adjacent paravertebral soft tissues, culminating in severe biomechanical instability. Although several cases of lumbosacral spondyloptosis have been documented, not many cases of traumatic lumbar spondyloptosis have been published in the literature.

**Case presentation:**

We present a case of a 34-year-old man of Nilo-Hamitic ethnicity who presented to our unit with paraplegia following injury from the collapse of a concrete wall. Radiographic images showed spondyloptosis at the fourth lumbar vertebral level. He underwent surgery where decompression, reduction, posterior instrumentation and bone grafting through a posterior approach were done. He started regaining motor power 48 hours postoperatively. He is currently undergoing rehabilitation and is steadily improving, 2 months postoperatively.

**Conclusions:**

In limited-resource settings there is a tendency of “skilful neglect” of complex injuries. Where resources allow, surgical reconstruction of spondyloptosis should be attempted irrespective of the severity of the initial neurological deficit because there are chances of neurological improvement.

## Introduction

Traumatic spondyloptosis is defined as 100% or greater subluxation of a superior vertebral body on an inferior one in the coronal or sagittal plane secondary to an injury [1]. Spondyloptosis results in one spinal segment being lodged in the anterior or posterior space of the adjacent segment [2]. It is the most severe of translation spine injuries and results in severe biomechanical instability caused by complete disruption of structural elements of the vertebral column and the adjacent paravertebral soft tissues [3]. Patients usually develop severe neurological injuries and are commonly classified as American Spinal Injury Association (ASIA) A. Although several cases of lumbosacral spondyloptosis have been documented, not many cases of traumatic lumbar spondyloptosis have been published in the literature [4–12]. We report a rare case of traumatic spondyloptosis at the fourth lumbar vertebra-fifth lumbar vertebra (L4-L5) level caused by the collapse of a concrete wall. The patient had reconstructive surgery and improved to ASIA D 2 months after surgery. We describe the clinical presentation and surgical management.

## Case presentation

**Figure 1:**
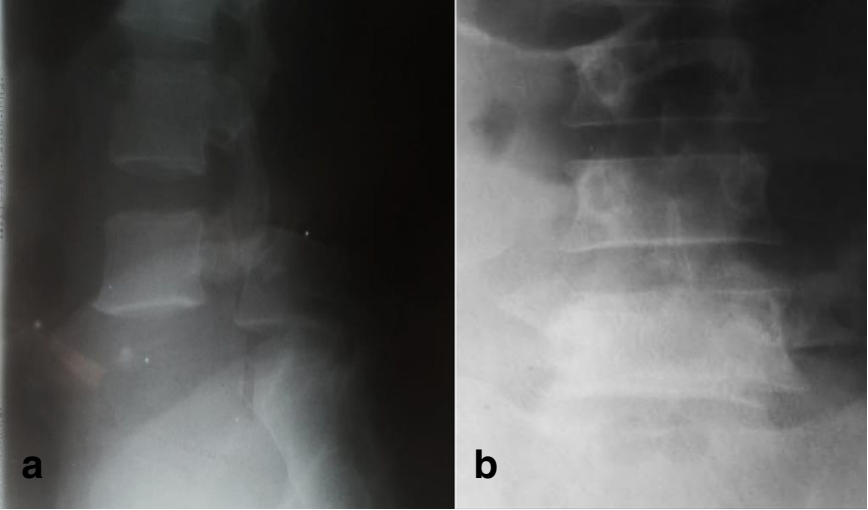
**Preoperative radiographs.** Plain anteroposterior **(a)** and lateral radiographs **(b)** of the lumbar spine showing spondyloptosis with L4 lying anterior to the L5 vertebral body in the sagittal plane.

A 38-year-old man of Nilo-Hamitic ethnicity was admitted to our spine unit with paraplegia following an injury to his lumbar spine. He was hit in the back while trying to escape from a collapsing concrete wall. He presented with severe lower back pain, inability to move his lower limbs, urine retention and altered sensation in his legs and feet. He was fully conscious, and had an obvious hyperlordotic deformity and bruising in his lumbar region. He had muscle power grade 0/5 and cutaneous sensory loss below the L3 level bilaterally. He had no perianal sensation and rectal tone was absent. We graded him as ASIA A. Plain radiographs (Figure 1) showed sagittal misalignment of his lumbar spine with complete anterior translation of L4 on L5. There also was a left transverse process fracture of L4. We had no access to computed tomography scans and magnetic resonance imaging.

**Figure 2:**
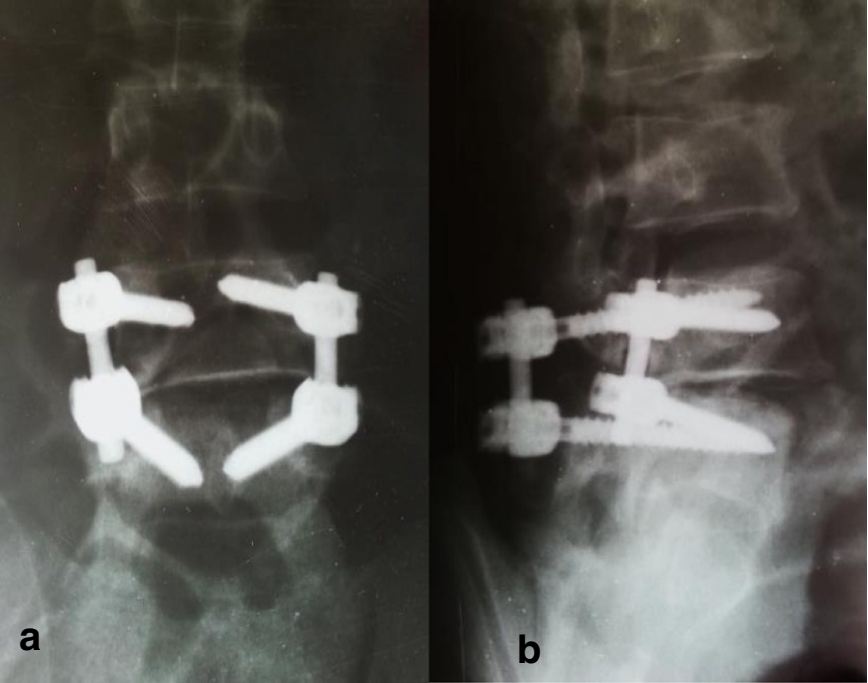
**Postoperative antero-posterior (a) and lateral (b) radiographs of the lumbar spine.** Good reduction is shown with short segment posterolateral fixation.

The patient was stabilized and had spine surgery 4 days after injury. He had no chronic diseases and this was his index surgery. He was positioned prone on a spine frame and his spine approached through a longitudinal midline incision, exposing L3-S1 vertebrae. We found: extensive contusion and disruption of his musculoligamentous structures; an obvious dislocation at the L4/5 level with disruption of the zygapophyseal joints; fractures of the pars, tip of the spinous process and left transverse process of the L4 vertebra; and cerebrospinal fluid leakage. Decompression of the neural elements was done first, through an L4 laminectomy. The thecal sac was found to have ruptured, but with no obvious injury to the cauda equina*.* Good reduction of the dislocation (Figure 2) was achieved through a process of distraction and leverage of the L4 vertebra using a laminar spreader and periosteal elevators. Posterolateral spinal fusion was then done using pedicle screw instrumentation. Pedicle screws were placed bilaterally into the L4 and L5 vertebral bodies using a free hand technique. Then, pre-bent titanium rods were placed in the screw heads and the screw caps tightened. A bone graft was then harvested from the right posterior iliac crest; and together with morselized bone obtained from the surgical site, the graft was packed into the lateral gutters. The dura was carefully repaired and wound closure done in the standard manner. There was no intraoperative fluoroscopic guidance or neurophysiological monitoring during the entire procedure.

The patient started regaining motor power 48 hours after surgery. Two months postoperatively, his hip flexion had improved to muscle power grade 4/5; knee extension, dorsiflexion of his feet and big toe had improved to grade 3/5; but dorsiflexion was still absent (grade 0/5). He also still had bladder dysfunction and was using a condom catheter. We graded him as ASIA D at the last follow-up. He is currently in a wheelchair and undergoing rehabilitation.

## Discussion

The management of lumbar spondyloptosis is challenging in resource-limited settings. Many of these complex injuries are usually left alone in developing countries where there are limited resources to purchase spine instrumentation systems and devices coupled with the lack of skilled surgeons to perform the surgeries [13]. However, conservative treatment is ineffective because of the resultant instability. Successful use of orthopaedic manoeuvres such as traction in late-stage spondylolisthesis [14] and manipulation under anaesthesia followed by internal fixation [15] have been described.

Surgical reconstruction gives the best outcome because it restores the stability of the vertebral column and facilitates rehabilitation. The one-stage short segment decompression, reduction, fixation, and fusion through a posterior approach used in our patient has been described before and with good results [16]. A posterior approach was used because it has fewer associated complications [17]. Other surgical options that have been documented particularly for chronic dislocations include, both instrumented and non-instrumented *in situ* fixation [18], and resection of the L5 vertebral body [19, 20]. Whenever possible, spondyloptosis should be reduced early to increase the chances of neurological recovery [21]. Neurologic recovery is variable and is highly dependent on the severity of injury. In this case neurological improvement was observed within 48 hours of surgery. It is very important to note that the prognosis of lumbar spondyloptosis depends more on the degree of anatomical injury found at surgery than on radiologic assessment of the injury [11].

## Conclusions

In limited-resource settings there is a tendency of “skilful neglect” of complex injuries. Where resources allow, surgical reconstruction of spondyloptosis should be attempted irrespective of the severity of the initial neurological deficit because there are chances of neurological improvement.

## Consent

Written informed consent was obtained from the patient for publication of this case report and accompanying images. A copy of the written consent is available for review by the Editor-in-Chief of this journal.

## Abbreviations

ASIA: American Spinal Injury Association
L4: Fourth lumbar vertebra
L5: Fifth lumbar vertebra.
